# Nature and Treatment of Piles

**Published:** 1907-08

**Authors:** 


### NATURE AND TREATMENT OF PILES.

Barker defines a pile as primarily a dilated or varicose condition of one or more of the plexus of veins lying under the mucous membrane of the last inch or so of the lower bowel. Probably there is a congenital weakness of the muscular coat of these veins in most cases. There is usually a history of haemorrhoids in other members of the family, and the trouble, as a rule, develops early in life. But there are other factors which divide themselves into two groups; first, any cause of obstruction to the return of blood through the inferior haemorrhoidal plexus; and, secondly, any cause of local irritation. In the first group come a slugish liver with engorgement of the veins of the portal system, heart disease, pregnancy, and most common of all, a loaded bowel in chronic constipation and with prolonged sitting or standing. In the second come every kind of septic irritation—want of cleanliness after defection, and local mechanical irritation, such as chafing, bumping, etc. Many cases of piles even when very large, do not require operation for their cure, and others, when first seen, are not in a state to permit of operation, although it will ultimately be necessary. The first step in treatment is to relieve any engorgement from within by gentle laxatives and bodily exercise. The piles and surrounding parts should be washed three times daily, first with soap and water, then with an antiseptic lotion—e. g., bichloride solution, one to one thousand. The piles should then be reduced within the annus if possible, but without force, and the patient should lie down for a while. This procedure should be carried out every morning, after the bowels have been moved, and at night just before going to bed. It often brings about a cure, and if not, it greatl yimproves the prospects for success of an operation. The writer is opposed to ligation or cauterization, and holds Whitehead's classical operation to be the best. Laxatives should be given four days before the operation, combined with enemata, in order to make sure that the bowel is empty. In order to control the anus properly during operation, it is well in all cases to stretch the sphincter with the thumbs. But if spinal analgesia is employed such stretching it not necessary.—Lancet, June 22nd, 1907.

